# A retrospective analysis of trends in primary knee arthroplasty in Germany from 2008 to 2018

**DOI:** 10.1038/s41598-021-84710-y

**Published:** 2021-03-04

**Authors:** Michael Worlicek, Matthias Koch, Popp Daniel, Viola Freigang, Peter Angele, Volker Alt, Maximilian Kerschbaum, Markus Rupp

**Affiliations:** 1 Department of Trauma Surgery, University Medical Centre Regensburg, Franz-Josef-Strauss-Allee 11, 93053 Regensburg, Germany; 2 Sporthopaedicum Regensburg, Regensburg, Germany

**Keywords:** Health care, Health care economics, Health policy

## Abstract

Unicompartimental and total knee arthroplasty is one of the most successful and most performed operations worldwide. In the last years the number of primary knee arthroplasty increased constantly. The aim of this study is to analyze the rising numbers of primary knee arthroplasty and to see how it is used in Germany. In this retrospective study data, provided by the Federal Statistical Office of Germany from 2008 to 2018 was analyzed, using operation codes from the German procedure classification system and characteristics like age, sex and type of the prosthesis. We found a slight increase of unicompartimental and total knee arthroplasty over the investigated 10 years from 150.504 in 2008 up to 168.479 procedures in 2018, with a maximum of 169.334 in 2017. Most patients were female and over 65 years old. Interestingly, there was an obvious decrease of regular TKA in the year 2013, with a relevant impact on the total number of procedures. In the following years the number rised again reaching the former level in 2015 and is still increasing. The highest increase was found in partial knee arthroplasty, with a constant rise every year, starting with 7988 in 2008 up to 21.072 in 2018. In contrast, we found a relevant reduction of constrained prosthesis in primary TKA, whereas the number of semi-constrained prosthesis in primary TKA is again rising after a decrease in 2015. We found that the number of bicondylar TKA and especially UKA increased from 2008 to 2018. Regarding an aging population, we can expect a rising number for Primary knee arthroplasty and in consequence a rising number of revision arthroplasty in the future. This will be a challenging cost factor for the healthcare system in Germany.

## Introduction

In Germany, primary total knee arthroplasty (TKA) is one of the most performed surgical procedures.

Due to an increased life expectancy and demographic changes with an increasing number of older patients having a high demand on performance, the number primary knee arthroplasty is rising constantly^1,2^.

Intriguingly, also, the number of younger patients receiving primary knee arthroplasty is rising in the western population^3–6^. This development leads to high costs for the healthcare systems and to a consecutive increasing number of revision arthroplasties, which are burdensome for the patients^7,8^.

Especially unicompartimental knee arthroplasty (UKA) has gained interested in the last decade. Reasons are suspected benefits such as less tissue trauma, reduced blood loss during surgery and better functionality due to faster rehabilitation, recovery and improved range of motion^9^.

In Scandinavia, England, Australia and Canada arthroplasty registries numbers of these procedures are listed in national arthroplasty registries^10–13^. In October 2011 the German arthroplasty register (EPRD = Endoprothesenregister Deutschland) was founded and since then collected data from arthoplasty procedures. But not all hospitals and surgeons send their data to the EPRD, so there might be a lack of numbers.

Estimations for the future development of primary knee arthroplasty in Germany over the next decades exist^7^. However, trends of different primary arthroplasty procedures during the recent years have to be elucidated. Therefore, aim of the study was to analyze the overall trend of different primary knee arthroplasty procedures. In addition, the influence of age and sex on primary knee arthroplasty procedures was investigated from 2008 through 2018.

## Materials and methods

Data from 2008 through 2018 was provided by the Federal Statistical Office of Germany (Destatis). This database includes all annual surgical procedures, based on operation and procedures codes (OPS) from all German hospitals and medical institutions. Since it is mandatory for all somatic German health care providers to settle up costs by the diagnosis related group system, all knee arthroplasty procedures were included to the provided data set. Destatis approved the use of data and there is no requirement of consent.

Ethical approval was not considered necessary by the Ethics Committee at the University of Regensburg since the study did not include identifiable human material or patient data. The study is in accordance with ethical standards of the local ethical committee.

A query of the database was performed for all patients who underwent Primary knee arthroplasty using the OPS code 5-822. Subgroup analysis was then performed depending on age and sex, for TKA and unicompartimental knee arthroplasty. Since a change in mostly all subgroups of the OPS codes was performed, subgroups before and after 2014 were adapted accordingly (see Table 1). Patellofemoral and bicompartimental arthroplasty procedures were excluded, due to their low volume in numbers. OPS codes 5-822.x0/x1/x2/y were also excluded since they stand for “other” and unspecified TKA which could not be allocated to a specific surgical procedure.

**Table 1:** OPS codes for the procedures carried out and analyzed.

OPS code 5-822	Description
00/01/02	Unicompartemental knee arthroplasty with/without cement and hybrid
10/11/12/20/21/22	Bicondylar TKA, non-constrained, including posterior stabilized, with/without patella resurfacing, with/without cement and hybrid; including extended bending ability
a1/a2/b1/b2/g0/g1/g2/j1/j2	TKA, semi-constrained with femoral and tibial stem, with/without patella resurfacing, with/without cement and hybrid
30/31/32/40/41/42	
h0/h1/h2	TKA, constrained, with/without patella resurfacing, with/without cement and hybrid, including special prosthesis like tumor prosthesis
60/61/62/70/71/72/90/91/92	

Data was analyzed and graphically displayed using the statistical software SPSS Version 26.0 (IBM, SPSS Inc. Armonk, NY, USA).

## Results

**Figure 1:**
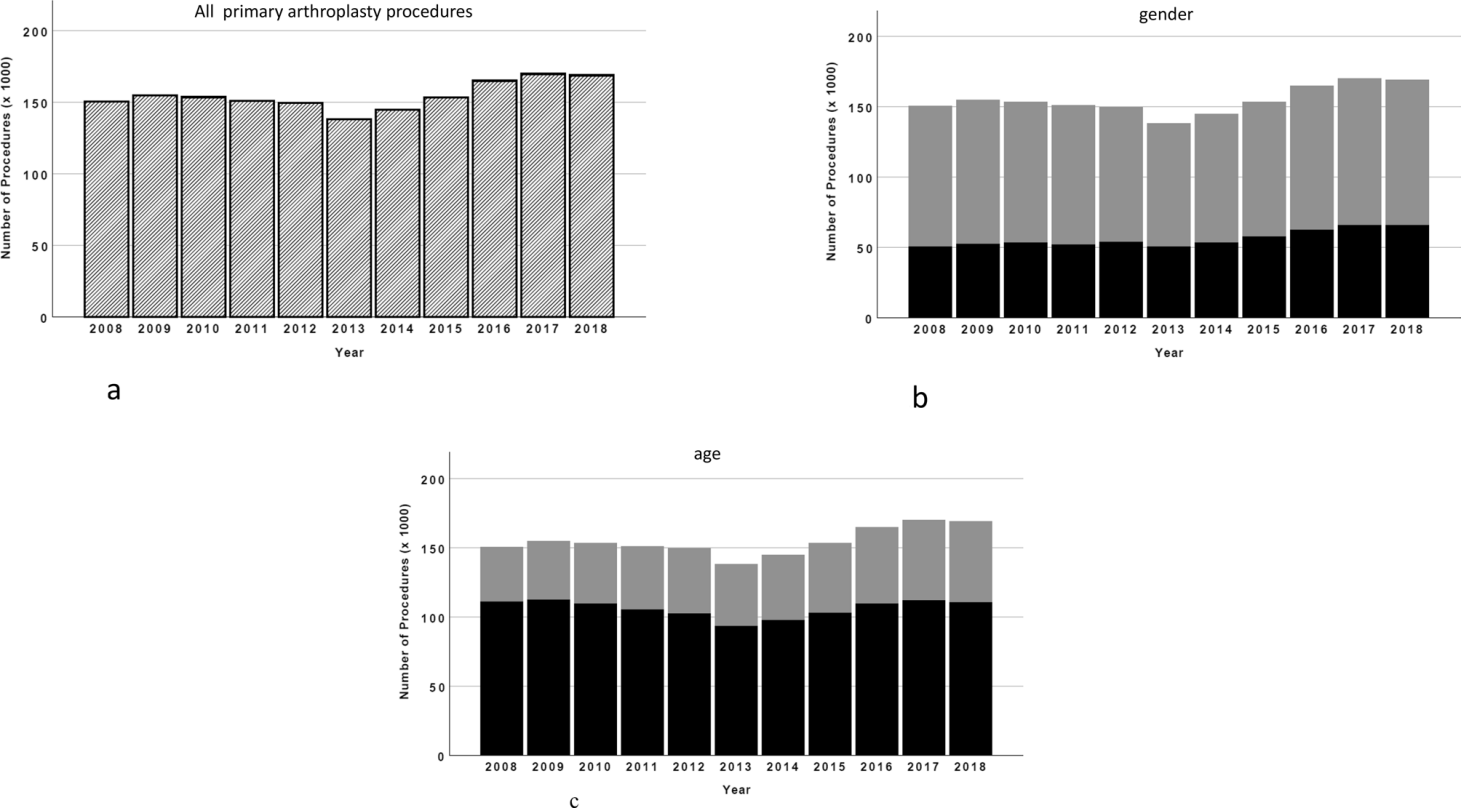
(**a**) All performed primary knee arthroplasty procedures from 2008 to 2018. (**b**) All performed primary knee arthroplasty procedures from 2008 to 2018, with regard on the gender of patients (grey = female; black = male). (**c**) All performed primary knee arthroplasty procedures from 2008 to 2018, with regard on the age of patients (grey: < 65 years; black ≥ 65 years).

A slight increase of partial and total knee arthroplasty was observed over the investigated 11-year period from 150,504 in 2008 up to 168,479 procedures in 2018, with a maximum of 169,334 in 2017 (Fig. 1a). This represents a total increase of 12.0%. Around two thirds of all procedures have been performed in female patients (Fig. 1b) and most patients were older than 65 years (Fig. 1c).

**Figure 2:**
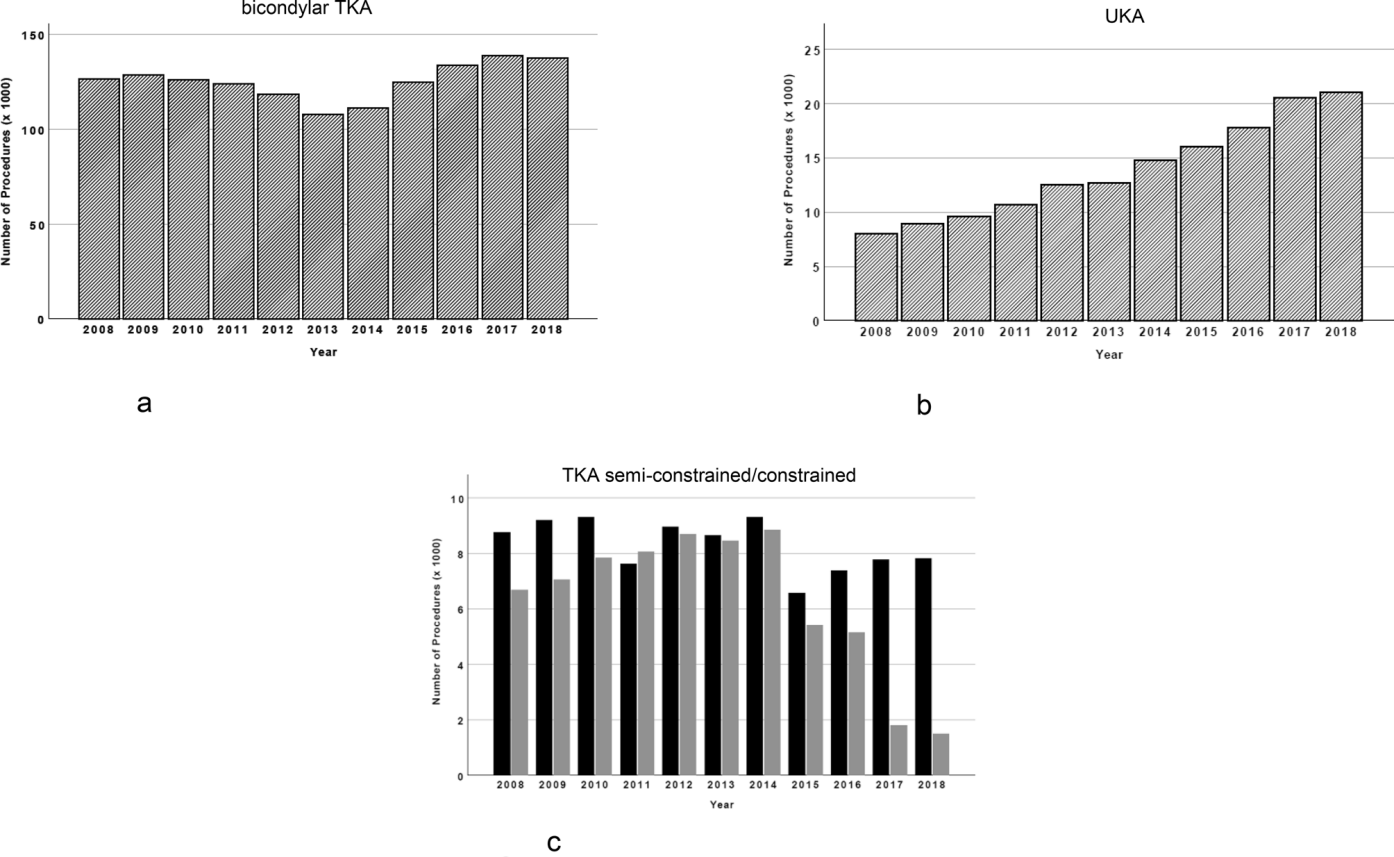
(**a**) All performed regular total knee arthroplasty procedures from 2008 to 2018. (**b**) All performed partial knee arthroplasty procedures from 2008 to 2018. (**c**) All performed semi-constrained and constrained total knee arthroplasty procedures from 2008 to 2018 (black = semi-constrained; grey = constrained).

Interestingly, there was an obvious decrease of bicondylar TKA in the years 2012 (− 4.5%) and 2013 (− 13.3%) compared to 2011, with a relevant impact on the total number of procedures (Figs. 1a, 2a). In the following years the number increased again reaching the former level in 2015 and has increased since then.

Regarding the analyzed subgroups, we found the highest increase in UKA, with a constant rise every year, starting with 7.988 in 2008 up to 21.072 in 2018 (Fig. 2b), which represents a total increase of about 164% in one decade. In contrast to the rising numbers of partial and bicondylar TKA, we found a relevant reduction of constrained prosthesis in primary knee arthroplasty, whereas the number of semi-constrained prosthesis in primary knee arthroplasty is again rising after a decrease in 2015. However, until 2018 it has not reached its maximum of 663 (Fig. 2c). But considering the high number of regular TKA in 2018 (137.726), the small number of 9305 constrained and semi-constrained prosthesis does not seem relevant (Fig. 2a,c).

The majority of patients, who underwent primary knee arthroplasty was female and minimum 65 years old. Over the observed period the distribution between male and female patients was always around one third to two thirds. Interestingly, the number of patients 65 years and older treated with primary knee arthroplasty did not change relevantly. Most of the growth of primary knee arthroplasty was related to patients younger than 65 years, from 39.595 in 2008, up to 57.947 in 2018, which represents a total increase of 46.3%.

## Discussion

Primary knee arthroplasty is one of the most performed surgical procedures in Germany^14^. In western industrial nations, the healthcare system is challenged by demographic changes with an aging and concomitant shrinking population. Due to this growing population and the claim to have a good quality of life, the number of primary knee arthroplasty seems to be rising every year. Until today, access to arthroplasty in Germany is unrestricted, but as average life expectancy of women and men in Germany is 84.1, and 79.1 years, respectively (Statistisches Bundesamt), the need for Primary knee arthroplasty and revision arthroplasty is a relevant cost for the healthcare system^7,8^. A recent study tried to predict the development of total hip and knee arthroplasty (THA/TKA) in Germany up to the year 2040. The data showed, that the number of THA and TKA will increase despite an expected decrease of the population^7^. To our knowledge, this is the first study, which gives an overview of the actual numbers of primary knee arthroplasty in Germany over a period of 11 years, considering subprocedures, age and sex.

Regarding all primary knee arthroplasty procedures in Germany from 2008 to 2018 we found a slight increase over the years, which is in accordance with studies from the UK, Italy and the United States^8,15,16^.

In the year 2012, there has been a medial discussion about the high number of performed elective surgeries with focus especially on THA and TKA. The former minister of health, Daniel Bahr (FDP), has critisized the increasing number of operations in hospitals and wanted to limit them. In an interview with the Rheinische Post published on May 1st 2012 he said: “Germany is considered the world champion in total knee and hip arthroplasty. Health insurance companies and experts doubt whether the increase in the number of cases is necessary”. He also criticized orthopedic surgeons who would perform surgical procedures for economic reasons.

We assume, that this medical campaign with its concerns caused in the German population, lead to the observed decrease of regular TKA in 2012 and especially one year later in 2013. Interestingly, procedures, such as partial, constrained and semi-constrained knee arthroplasty, were not affected by this medial discussion. A further increase of TKA numbers in the following years could be observed as well.

During the observed 11-year period the number of UKA was rising constantly. This might be due to the further development of implants and instruments and better surgical techniques^17^. Several studies demonstrated UKA being a less invasive, more cost-effective alternative to TKA, if properly indicated and patient selection criteria are respected^18–20^. A typical indication for UKA is monocompartimental osteoarthritis after a former complete or partial meniscectomy, as it was regularly performed starting in the 1970s up to the early 1990s, especially in young patients after sport injuries^21–24^.

Meanwhile, several studies showed that especially complete meniscectomy leads to cartilage damage and osteoarthritis in the compartiment, caused by a deviation of the leg axis and a higher pressure on the cartilage^25–27^. In consequence, complete meniscectomy is a rare operation today. Nevertheless, there is still a relevant number of patients, who underwent meniscal damage and surgery. This patient cohort suffering from unicompartimental osteoarthritis might be an additional reason for the rising number of UKA, until today. Another reason for the increasing number of UKA, especially after 2016, could be in connection with a new regulation of statutory health insurance funds in Germany that came into force this year. Based on the work of Sihovnen et al.^28^, which casts doubt on the effectiveness of arthroscopic operations in degenerative meniscus tears, it was decided to stop remunerating this type of operation. This may have led to an increased indication position for UKA. This is reflected in a significant increase in UKA after 2016 (Fig. 2b).

Another interesting finding of this study was, that the majority of patients with TKA was female. The number of female patients has always been twice as high, compared to male patients during the observed period. This result is in accordance with former studies, which showed a higher incidence for osteoarthritis of the knee in female patients^29,30^. The reason for this unequal distribution seems to be multifactorial and should be investigated in future studies.

As expected, the majority of patients with TKA is over 65 years old and the number of these patients did not change relevantly. But we found a constant increase of patients younger than 65 years, who underwent primary knee arthroplasty during the observed period. This is in accordance with several studies, which predict a relevant increase of younger patients needing primary knee arthroplasty^5,31–33^. A major task, which will occupy us in the future with regard on revision surgery.

In contrast to the rising number of UKA and regular TKA, we found an obvious reduction of constrained TKA as well as a temporary decrease of semi-constrained TKA. This might be again due to the further development of implants and instruments and better surgical techniques, but needs to be investigated in future studies.

This study has several limitations. This is an evaluation of historical data provided by Destatis, so there might be a lack of captured numbers. As mentioned above, we excluded the OPS codes 5-822.x0/x1/x2/y, as well as patellofemoral and bicompartimental arthroplasty, so not all arthroplastic procedures were analyzed, but because of the small number of this operations, this seems negligible. Finally, we only analyzed primary knee arthroplasty procedures, revision arthroplasty has not been part of this study.

## Conclusion

We found that the number of bicondylar TKA and especially UKA increased from 2008 to 2018. Regarding an aging population, we can expect a rising number for Primary knee arthroplasty and in consequence a rising number of revision arthroplasty in the future. This will be a challenging cost factor for the healthcare system in Germany.
